# Correlation of Serum Biomarkers and Magnetic Resonance Spectroscopy in Monitoring Disease Progression in Patients With Mitochondrial Encephalomyopathy, Lactic Acidosis, and Stroke-Like Episodes Due to mtDNA A3243G Mutation

**DOI:** 10.3389/fneur.2018.00621

**Published:** 2018-07-27

**Authors:** Ha Neul Lee, Choon-Sik Yoon, Young-Mock Lee

**Affiliations:** ^1^Department of Pediatrics, Yonsei University College of Medicine, Seoul, South Korea; ^2^Department of Diagnostic Radiology, Yonsei University College of Medicine, Seoul, South Korea

**Keywords:** lactate peak, magnetic resonance spectroscopy (MRS), mitochondrial encephalomyopathy with lactic acidosis and stroke-like episodes (MELAS), mitochondrion, pediatric, serum lactate

## Abstract

**Background:** Analysis of serum biomarkers and magnetic resonance spectroscopy (MRS) are useful for monitoring disease progression in patients with mitochondrial encephalomyopathy, lactic acidosis, and stroke-like episodes (MELAS). We evaluated the correlation of serum biomarkers and MRS parameters during changes associated with stroke-like episodes.

**Methods:** In 13 symptomatic MELAS patients carrying the A3243G mutation, we retrospectively obtained 207 voxels from 41 MRS studies, which were divided into three groups according to the temporal association with stroke-like episodes. The MRS NAA/Cr, Cho/Cr, NAA/Cho ratios, the presence of a lactate peak, serum biomarkers, serum lactate level and the pyruvate (Lac/Pyr) ratio were determined.

**Results:** In regions with acute infarcts, the severity of serum Lac/Pyr and that of the MRS lactate peak (*P* = 0.0007) correlated; serum lactate (*P* = 0.02), severity of elevated serum lactate (*P* = 0.04), and serum Lac/Pyr (*P* = 0.02) correlated weakly. In previously infarcted regions, the severity of the MRS lactate peak and serum Lac/Pyr (*P* = 0.03), as well as the severity of serum Lac/Pyr (*P* = 0.02) were weakly correlated. In structurally normal regions, we found a weak to moderate negative correlation between serum lactate and MRS NAA/Cr (*P* = 0.008), and between the severity of elevated serum lactate and MRS NAA/Cr (*P* = 0.002) as well as MRS NAA/Cho (*P* = 0.02).

**Conclusions:** MRS parameters correlate with specific serum biomarkers, and are useful for monitoring changes in brain metabolites, particularly as related to stroke-like episodes.

## Introduction

Mitochondrial encephalomyopathy, lactic acidosis, and stroke-like episodes (MELAS) syndrome is characterized by its unique features of stroke-like episodes and associated neurological symptoms and psychomotor regression (1, 2), related to defective mitochondrial energy metabolism (3, 4). Biochemically, acute metabolic derangement in mitochondrial disease presents with serum lactic acidosis, reflecting overall mitochondrial dysfunction (3, 5, 6), and sometimes with an elevated serum lactate-to-pyruvate (Lac/Pyr) ratio, which indicates impairment of the mitochondrial respiratory chain enzyme complex (3, 7). For these reasons, biochemical analysis of serum lactate, pyruvate, and the Lac/Pyr ratio is commonly used to identify patients suspected of having mitochondrial diseases. Moreover, obtaining biomarker data from serum is less invasive than other procedures, such as muscle biopsy. Serum biomarker analysis is an easily available option for monitoring the clinical progress of patients with mitochondrial diseases (3, 5, 7), including patients with MELAS.

According to the recent diagnostic criteria reported by Yatsuga et al. (8), acute focal lesions seen on brain images, such as magnetic resonance imaging (MRI) and magnetic resonance spectroscopy (MRS), are common in MELAS. These changes occur in relation to stroke-like episodes and vary, but occur outside the usual territories of vascular infarction (1, 9–12). MRS is a noninvasive tool for evaluating brain metabolites, including choline (Cho), creatine (Cr), *N*-acetylaspartate (NAA), and lactate peaks. Previous studies have confirmed that there is a decrease in NAA) (9–15), NAA/Cr, and Cho/Cr (5, 13, 14), and that a lactate peak is present in lesions associated with stroke-like episodes, and sometimes even in normal-appearing regions on MRI scans (1, 9–16). Previous MRS studies in MELAS patients and MELAS carriers initially interpreted the lactate peak in comparison to conventional MRI data (1, 9–12) and have advanced to interpretation of several different metabolite concentrations qualitatively or quantitatively, sometimes in relation to certain treatment options (14, 17), functional changes (4, 13), or even stroke-like episodes (15, 16). However, different study populations of mitochondrial disorders, methods, and MRS measurements, were involved across studies (5, 18). Additionally, although there is a correlation between cerebral lactic acidosis and functional neurological impairment (13) and cerebral lactic acidosis has even been applied for predicting risk of symptom development in obligate carriers (4), the significance of serum lactic acidosis in relation to MRS metabolite changes remains to be elucidated.

There have been no reports on serial MRS metabolite changes associated with serum biomarkers, particularly in temporal relation to clinical stroke-like episodes. Therefore, we assessed the correlation of these parameters, to verify whether the relatively non-invasive tools of serum biomarker analysis and MRS parameters could reflect changes associated with stroke-like episodes, and their relationship. We aimed to determine which specific serum biomarkers and MRS parameters would best reflect disease progression and allow prediction of patient prognoses.

## Materials and methods

### Patient selection and clinical data collection

Patients who were symptomatic, and who had clinical pictures compatible with the mitochondrial disease criteria proposed by Bernier et al. (19) were retrospectively recruited from the electronic medical records of Gangnam Severance Hospital. From this pool, patients in whom a diagnosis of MELAS was confirmed, based on the diagnostic criteria reported by Yatsuga et al. (8), were chosen, with a follow-up period from March 2006 to January 2017. Among the 22 patients meeting the diagnostic criteria for MELAS, 13 patients who were positive for the A3243G mutation and for whom MRS data were available were selected to ensure a more homogeneous study population.

Intensive evaluations were performed on these subjects. Laboratory test results, including serum lactic acid, pyruvic acid, and amino acid levels, and urine organic acids, were obtained concurrent with MRS examinations. Clinical data were also obtained, including sex, age of initial symptoms, age of confirmative diagnosis of MELAS, initial presenting symptoms, age at the last visit, and associated organ system involvement.

The study was approved by the Institutional Review Board of Gangnam Severance Hospital, Yonsei University College of Medicine. We also obtained informed consent from the patients and the families for analysis of serum and imaging data obtained.

### MRS data collection in MELAS patients

**Figure 1:**
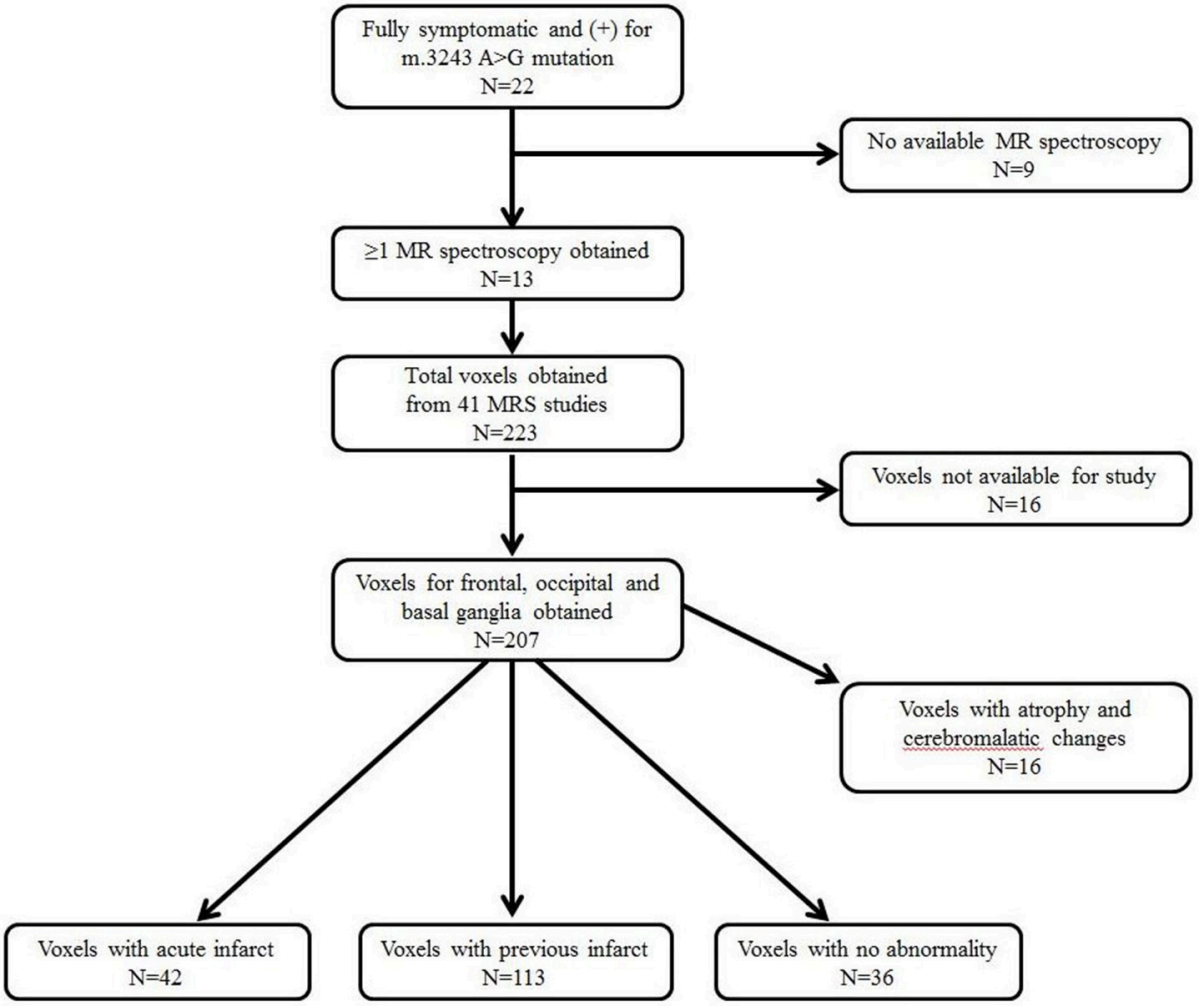
Selection of patients and magnetic resonance spectroscopy (MRS) data.

**Figure 2:**
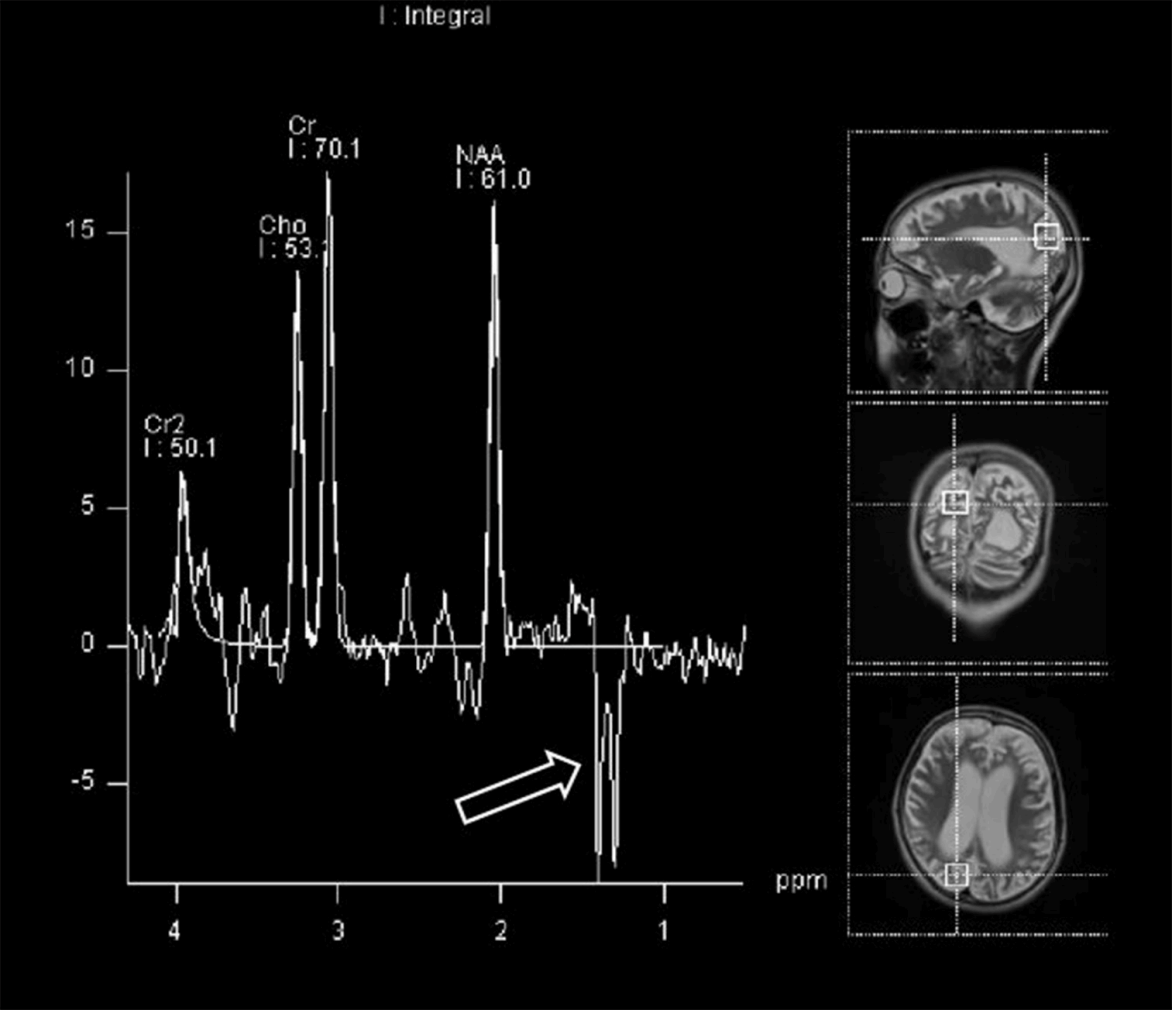
Magnetic resonance spectroscopy (MRS) demonstrating lactate peak (open arrow).

Further data, obtained from brain MRI and MRS studies, were also collected. Figure 1 shows the details on how we selected appropriate MRS data for review. ^1^H-MRS was performed on a GE 750W 3.0T scanner with a 16-channel head-neck combined coil after the conventional magnetic resonance imaging examination. MR imaging (MRI) included T2 weighted and 2D fast spine echo scans. The patients laid flat on the examining table with their heads fixed during the scanning. For some of the patients, but not all, were sedated using chloral hydrate, midazolam or ketamine based on the anesthesiologist and the pediatrician's decision following consistent sedation anesthesia protocol at our institute. Single voxel spectroscopy (PRESS, Repetition time = 1,500 ms, Echo time = 144 ms, Acquisition time = 3.48 min, voxel = 20 × 20 × 20 mm) was used. The voxel was placed by an MR imaging technician under the supervision of a neuroradiologist, who has around 30 years of experience in this field of radiology. The regions of interest were placed in the frontal and occipital gray matter and basal ganglia of bilateral hemisphere. A rapid automated shimming method was conducted by the machine. After water suppression and tuning, ^1^H-MRS spectra were obtained and directly analyzed by Functool 9.4.05 software in the GE Medical System. All data were processed using a Gaussian line and were read by two separate radiologists. Known chemical shifts, including lactate at 1.3 ppm, NAA at 2.02 ppm, Cr at 3.05 ppm, and Cho at 3.22 ppm, were reviewed. Ratios for NAA/Cr, Cho/Cr, and NAA/Cho were evaluated, as well as the presence of the lactate peak. The height of lactate peak was further categorized per readings of two separate neuroradiologists (Figure 2).

Among the 22 patients with a diagnosis of MELAS followed at our institution, 13 patients positive for the A3243G mutation and with ≥1 MRS evaluation were recruited; this included a total of 41 MRS studies (Figure 1). A total of 207 voxels were obtained from the frontal and occipital regions, and the basal ganglia, which were suitable for the study. We excluded 16 voxels related to atrophic and cerebromalatic changes that were not associated with clinical stroke-like episodes, and then divided the data into three groups: voxels associated with an acute infarct (*N* = 42), voxels associated with a previous infarct (*N* = 113), and voxels with no abnormal findings, i.e., normal MRI regions (*N* = 36). The definitions of acute and previous infarcts were as follows. For acute infarcts, the MRS was obtained within 1 week of clinical stroke-like episodes and showed findings positive for new structural changes. For chronic infarcts, MRS was obtained at least 6 months after a previous clinical stroke-like episode, with no additional clinical stroke-like episode during the interval, and with no new structural changes visible.

### Correlation of serum biomarkers and MRS parameters in association with stroke-like episodes

We also investigated the correlation of serum biomarkers with MRS parameters. The below-mentioned severity grading was arbitrarily defined, in accordance to our previous publications (2, 17). The serum biomarkers were obtained on the same day with MRS examinations, more specifically within ±1–2 h of the MRS examinations.

The severity of serum lactic acidosis was defined as follows: 0, normal (0–2 mmol/L); 1, ca. 2-fold of reference value (2–4 mmol/L); 2, ca. 3-fold of reference value (4–6 mmol/L); 3, ca. 4-fold reference value (6–8 mmol/L); and 4, >4-fold of reference value (>8 mmol/L). The severity of the serum Lac/Pyr ratio was defined as follows: 0, <20; and 1, ≥20.

The severity of the MRS region was defined as follows: 0, normal; 1, region with acute infarct; 2, region with a previous infarct. The severity of the lactate peak in MRS data was defined as follows: 0, no presence of a lactate peak; 1, mild presence of a lactate peak; 2, definite presence of a lactate peak.

### Statistical analysis

All analyses were performed using SPSS version 23.0 (SPSS Inc. Chicago, IL, USA). Descriptive statistics, including the mean, standard deviation, median, and range, were determined. The association between the serum biomarkers and MRS parameters was evaluated using the Spearman rank-correlation method. The correlation coefficient (*r*) was interpreted as follows (20): 0–0.2 and −0.2 to 0, no or very weak association; 0.2–0.4 and −0.4 to −0.2, weak association, and 0.4–0.6 and −0.6 to −0.4, moderate association. *P*-values less than 0.05 were considered statistically significant.

## Results

### Patient's characteristics

A total of 13 patients (six male and seven female subjects; Table 1) were recruited. All subjects were symptomatic, and each subject met the clinical diagnostic criteria reported by Yatsuga et al. (8) Their mean age at the appearance of the first symptom was 15.2 ± 9.4 years (range 4.6–37.3 years). MELAS diagnosis was confirmed at a mean age of 17.4 ± 10.3 years (range 6.3–37.5 years), and these subjects were followed until a mean age of 23.8 ± 8.0 years (range 15.5–42.9 years). MRS examinations were obtained at a mean age of 16.0 ± 14.2 (6.4–40.9) years. Subjects initially presented with various symptoms, all related to neurological issues. All 13 patients (100%) had central nervous system involvement, in addition to other systemic involvement (Table 1).

**Table 1:** Patient characteristics.

**Characteristics**	***N* = 13**
Sex (male: female)	6:7
Age of symptom onset (years)	15.2 ± 9.4 (4.6–37.3)
Age of MELAS diagnosis (years)	17.4 ± 10.3 (6.3–37.5)
Age at the last visit (years)	23.8 ± 8.0 (15.5–42.9)
Age at MRS examinations (years)	16.0 ± 14.2 (6.4–40.9)
**Initial presenting symptom (*****n*****, %)**	
Seizure	7 (53.8%)
Headache	4 (30.8%)
Visual disturbance	2 (15.4%)
Ataxia	1 (7.7%)
**Organs involved at the last visit (*****n*****, %)**	
Central nervous system	13 (100%)
Hearing impairment	11 (84.6%)
Cardiomyopathy	10 (76.9%)
Diabetes mellitus/Glucose intolerance	9 (69.2%)
Skeletal muscle weakness	7 (53.8%)
Visual disturbance	7 (53.8%)
Psychological issues	7 (53.8%)
Arrhythmia	5 (38.5%)
Gastrointestinal system	5 (38.5%)
Renal system	4 (30.8%)

### Correlation of serum biomarkers and MRS parameters in the total voxels

Altogether, 191 voxels were analyzed, irrespective of whether they had a temporal association with stroke-like episodes; using these voxels, we evaluated the correlation between serum biomarkers and MRS parameters by using Spearman's rank correlation test. Given that the correlation coefficient (*r*) is considered to reflect a stronger relationship when the regression value is closer to 1 or −1, we reviewed the grade of the correlation coefficient (*r*) even when there was a significant *P*-value of <0.05. Using these criteria, we could only find a weak positive correlation between the serum Lac/Pyr ratio and the severity of the MRS lactate peak (*r* = 0.252, *P* = 0.0004) and the severity of the serum Lac/Pyr ratio and the severity of the MRS lactate peak (*r* = 0.245, *P* = 0.0006), respectively (Table 2). We did not find any statistically significant correlation among the other parameters (Table 2).

**Table 2:** Correlation of serum biomarkers and magnetic resonance spectroscopy (MRS) parameters in total voxels (*n* = 191).

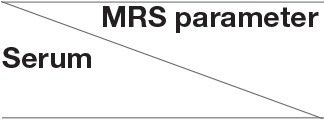		**Cho/Cr**	**NAA/Cr**	**NAA/Cho**	**Severity of lactate peak°**
Lactate	*r*	0.064	−0.180	−0.190	0.115
	*P*	0.38	0.01^*^	0.009^*^	0.11
Severity of elevated serum lactate^†^	*r*	0.006	−0.122	−0.113	0.083
	*P*	0.93	0.09	0.12	0.25
Lac/Pyr	*r*	−0.012	−0.143	−0.087	0.252
	*P*	0.86	0.005^*^	0.23	0.0004^*^
Severity of Lac/Pyr^§^	*r*	0.024	−0.182	−0.127	0.245
	*P*	0.74	0.01^*^	0.08	0.0006^*^

*Spearman correlation tests were performed for these analyses*.

* *P-values less than 0.05 were considered statistically significant*.

† *Severity of lactic acidosis was defined as follows: 0, normal (0–2 mmol/L); 1, ca. 2-fold; 2, ca. 3-fold; 3, ca. 4-fold; 4, >4-fold*.

§ *Severity of lactate/pyruvate was defined as follows: 0, <20; 1, ≥20*.

° *Severity of the lactate peak was defined as follows: 0, no presence of a lactate peak; 1, mild presence of a lactate peak; 2, definite presence of a lactate peak*.

*Lac/Pyr, lactate/pyruvate ratio; Cho/Cr, choline/creatine ratio; NAA/Cr, N-acetylaspartate/creatine ratio; NAA/Cho, N-acetylaspartate/choline ratio*.

### Correlation of serum biomarkers and MRS parameters in regions with acute infarcts

To evaluate whether serum biomarkers and MRS parameters correlate during the acute stroke-like episodes, we studied 42 voxels representing structural changes that were seen in association with clinical symptoms (Table 3). We did not find any statistically significant correlation between serum biomarkers and MRS Cho/Cr, NAA/Cr, and NAA/Cho ratio, besides the weak negative correlation between the severity of the serum Lac/Pyr ratio and the NAA/Cr ratio (*r* = −0.330, *P* = 0.03). However, we found a statistically significant association between serum biomarkers and the severity of the MRS lactate peak. The correlation was strongest between the severity of serum Lac/Pyr and the severity of the MRS lactate peak, although this was a moderately positive correlation (*r* = 0.503, *P* = 0.0007). Correlation was weakly positive for serum lactate (*r* = 0.361, *P* = 0.02), the severity of elevated serum lactate (*r* = 0.318, *P* = 0.04), and the serum Lac/Pyr ratio (*r* = 0.365, *P* = 0.02) with the severity of the MRS lactate peak.

**Table 3:** Correlation of serum biomarkers and magnetic resonance spectroscopy (MRS) parameters in regions with acute infarct (*n* = 42).

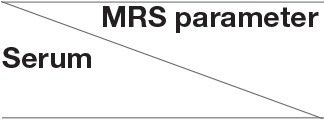		**Cho/Cr**	**NAA/Cr**	**NAA/Cho**	**Severity of lactate peak°**
Lactate	*r*	−0.157	−0.187	−0.118	0.361
	*P*	0.32	0.24	0.46	0.02^*^
Severity of elevated serum lactate^†^	*r*	−0.154	−0.145	−0.051	0.318
	*P*	0.33	0.36	0.75	0.04^*^
Lac/Pyr	*r*	−0.183	−0.237	−0.109	0.365
	*P*	0.25	0.13	0.49	0.02^*^
Severity of Lac/Pyr^§^	*r*	0	−0.330	−0.283	0.503
	*P*	1	0.03^*^	0.07	0.0007^*^

*Spearman correlation tests were performed for these analyses*.

* *P-values less than 0.05 were considered statistically significant*.

† *Severity of lactic acidosis was defined as follows: 0, normal (0–2 mmol/L); 1, ca. 2-fold; 2, ca. 3-fold; 3, ca. 4-fold; 4, >4-fold*.

§ *Severity of lactate/pyruvate was defined as follows: 0, < 20; 1, ≥20*.

° *Severity of the lactate peak was defined as follows: 0, no presence of a lactate peak; 1, mild presence of a lactate peak; 2, definite presence of a lactate peak*.

*Lac/Pyr, lactate/pyruvate ratio; Cho/Cr, choline/creatine ratio; NAA/Cr, N-acetylaspartate/creatine ratio; NAA/Cho, N-acetylaspartate/choline ratio*.

### Correlation of serum biomarkers and MRS parameters in regions with previous infarcts

To evaluate whether serum biomarkers and MRS parameters correlated during the chronic stage, we studied 113 voxels with evidence of previous infarcts, but that were not associated with new acute stroke-like episodes. Once again, we did not find a statistically significant correlation between serum biomarkers and MRS Cho/Cr, NAA/Cr, or NAA/Cho (Table 4). A statistically significant *P*-value (0.04) was found for the correlation between serum lactate and the Cho/Cr ratio; however, the correlation coefficient (0.196) was considered to reflect a very weak association. There were also statistically significant weak correlations between the serum Lac/Pyr ratio and the severity of the MRS lactate peak (*r* = 0.203, *P* = 0.03), and between the severity of the serum Lac/Pyr ratio and the severity of the MRS lactate peak (*r* = 0.213, *P* = 0.02; Table 4).

**Table 4:** Correlation of serum biomarkers and magnetic resonance spectroscopy (MRS) parameters in regions with previous infarcts (*n* = 113).

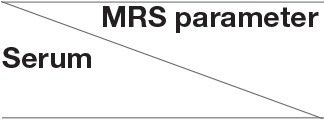		**Cho/Cr**	**NAA/Cr**	**NAA/Cho**	**Severity of lactate peak°**
Lactate	*r*	0.196	−0.048	−0.133	−0.013
	*P*	0.04^*^	0.64	0.16	0.89
Severity of elevated serum lactate^†^	*r*	0.103	0.081	0.007	−0.054
	*P*	0.28	0.39	0.94	0.57
Lac/Pyr	*r*	0.059	−0.044	−0.046	0.203
	*P*	0.54	0.65	0.63	0.03^*^
Severity of Lac/Pyr^§^	*r*	0.097	−0.127	−0.110	0.213
	*P*	0.31	0.18	0.25	0.02^*^

*Spearman correlation tests were performed for these analyses*.

* *P-values less than 0.05 were considered statistically significant*.

† *Severity of lactic acidosis was defined as follows: 0, normal (0–2 mmol/L); 1, ca. 2-fold; 2, ca. 3-fold; 3, ca. 4-fold; 4, >4-fold*.

§ *Severity of lactate/pyruvate was defined as follows: 0, < 20; 1, ≥20*.

° *Severity of the lactate peak was defined as follows: 0, no presence of a lactate peak; 1, mild presence of a lactate peak; 2, definite presence of a lactate peak*.

*Lac/Pyr, lactate/pyruvate ratio; Cho/Cr, choline/creatine ratio; NAA/Cr, N-acetylaspartate/creatine ratio; NAA/Cho, N-acetylaspartate/choline ratio*.

### Correlation of serum biomarkers and MRS parameters in normal regions

To evaluate whether serum biomarkers and MRS parameters correlated in the clinically silent stage, we studied 36 voxels reflecting no structural changes on MRI. In contrast to previous results, we did not find a statistically significant correlation between serum biomarkers and the severity of the MRS lactate peak (Table 5). However, we found a statistically significant weak to moderate negative correlation between serum lactate and the MRS NAA/Cr ratio (*r* = −0.437, *P* = 0.008), the severity of elevated serum lactate and the MRS NAA/Cr ratio (*r* = −0.501, *P* = 0.002), and the severity of elevated serum lactate and the MRS NAA/Cho ratio (*r* = −0.386, *P* = 0.02), respectively.

**Table 5:** Correlation of serum biomarkers and magnetic resonance spectroscopy (MRS) parameters in normal regions (*n* = 36).

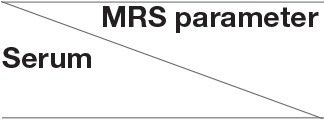		**Cho/Cr**	**NAA/Cr**	**NAA/Cho**	**Severity of lactate peak°**
Lactate	*r*	−0.048	−0.437	−0.292	0.005
	*P*	0.78	0.008^*^	0.08	0.98
Severity of elevated serum lactate^†^	*r*	−0.055	−0.501	−0.386	−0.033
	*P*	0.75	0.002^*^	0.02^*^	0.85
Lac/Pyr	*r*	−0.07	−0.173	−0.010	0.272
	*P*	0.68	0.31	0.95	0.11
Severity of Lac/Pyr^§^	*r*	−0.21	−0.123	0.085	0.241
	*P*	0.22	0.47	0.62	0.16

*Spearman correlation tests were performed for these analyses*.

* *P-values less than 0.05 were considered statistically significant*.

† *Severity of lactic acidosis was defined as follows: 0, normal (0–2 mmol/L); 1, ca. 2-fold; 2, ca. 3-fold; 3, ca. 4-fold; 4, >4-fold*.

§ *Severity of lactate/pyruvate was defined as follows: 0, < 20; 1, ≥20*.

° *Severity of the lactate peak was defined as follows: 0, no presence of a lactate peak; 1, mild presence of a lactate peak; 2, definite presence of a lactate peak*.

*Lac/Pyr, lactate/pyruvate ratio; Cho/Cr, choline/creatine ratio; NAA/Cr, N-acetylaspartate/creatine ratio; NAA/Cho, N-acetylaspartate/choline ratio*.

## Discussion

The present study showed that elevated lactate and the Lac/Pyr ratio positively correlate with the MRS lactate peak in regions demonstrating stroke-like episodes. These associations are stronger and more obvious during the acute stage than during the chronic stage. However, the importance of the MRS lactate peak seems to decrease in normal regions without evidence of stroke-like episodes, and serum biomarkers correlate negatively with NAA-associated MRS metabolite changes, including NAA/Cr and NAA/Cho. Serum and imaging data have not previously been compared simultaneously in a homogenous population of A3243G mutation-positive, symptomatic MELAS patients, particularly not in a temporal relationship to stroke-like episodes. Here, we have provided preliminary evidence that tracking the changes of both serum and MRS biomarkers may be useful for monitoring disease progression in MELAS patients.

Stroke-like episodes are cardinal symptoms of the MELAS syndrome (1, 18), and these episodes themselves present with numerous symptoms, eventually resulting in neurological deficits (1, 8, 21, 22). As shown in the present study, the serum Lac/Pyr ratio and its severity showed a statistically significant, but weak positive correlation, with the severity of the MRS peak when considering all the voxels, regardless of the timeline. However, we found a stronger and more distinct pattern of associations between serum biomarkers and MRS parameters when the temporal relationship with the stroke-like episodes was taken into consideration. This finding emphasizes the importance of the time-factor in the analysis of these data.

In regions involved in the acute stage of infarction, our data showed a statistically significant and novel association between serum biomarkers and the severity of the MRS lactate peak, with weak to moderate positive correlation. Our study data also supported previous literature that showed that the presence of the lactate peak in MRS is commonly associated with stroke-like episodes. As serum lactic acidosis represents overall mitochondrial dysfunction (3, 5, 6), and the Lac/Pyr ratio more specifically represents defective respiratory chain enzyme activity (3, 7) our results suggest that, during the acute stage of clinical stroke-like episodes, both mitochondrial dysfunction and respiratory chain enzyme defects are present and the degree of dysfunction correlates with the severity of the lactate peak on MRS. Thus, when a patient presents with stroke-like episodes, serum lactic acidosis, the Lac/Pyr ratio, and the severity of the latter may reflect changes in MRS, even when MRS may not be available because of the patient's unstable condition.

In the chronic stage, when clinical stroke-like episodes had occurred previously, without further acute changes for at least 6 months, the results were different from those obtained during the acute stage. In the chronic stage, only the serum Lac/Pyr ratio showed a statistically significant weak positive correlation with the severity of the MRS lactate peak, whereas serum lactic acidosis did not. It is not clear whether the smaller correlation coefficient, as compared to the acute stage, truly depicts a weaker correlation, given that our sample size was small; however, we may assume that the importance of the MRS lactate peak is less obvious in the chronic than in the acute stage. In addition, although the results were not statistically significant, we observed that the correlation between the serum lactate and the MRS lactate peak changed from a positive to a negative trend when compared to the acute stage, when the correlation between the serum Lac/Pyr ratio and the severity of the MRS lactate peak continued to show a positive trend. From this perspective, we may suppose that, after the patients suffer from acute stroke-like episodes, the overall mitochondrial dysfunction recovers faster, while the respiratory chain enzyme defect remained during the recovery phase. Yet, this hypothesis should be readdressed in future studies, with more study subjects, with an elaborate study design, in order to eliminate biases.

Our observations in regions with no structural changes during a clinically stable stage was in agreement with the findings of previous studies that reported that reduced NAA concentrations are common in MRS of MELAS patients, and represents ongoing neuronal damage, even in asymptomatic phases, and that these findings are not only restricted to structural regions (1, 9–16). Unlike results from the acute and chronic stage regions, we did not find significant correlations between serum biomarkers and the MRS lactate peak in these regions, which suggests that the clinical significance of the MRS lactate peak becomes less and that serum biomarkers do not accurately reflect the changes during the clinically stable stage, when there is no structural change. On the other hand, we noted that significant, negative associations were only found between the serum lactate and the MRS NAA/Cr and NAA/Cho ratios. As a decrease in NAA is considered to be a marker of disturbed neuronal integrity and of mitochondrial dysfunction, the present data seemed quite reasonable. We have also observed that the serum lactate correlates significantly with the MRS NAA-related biomarkers, while the serum Lac/Pyr ratio does not. This may indicate that the damage to overall mitochondrial function starts early, even during the clinically silent stage; however, direct damage to the respiratory chain enzyme happens later, with the strong metabolic disturbances of clinical stroke-like episodes.

Lastly, even though we analyzed the various MRS metabolite ratios, including Cho/Cr, NAA/Cr, and NAA/Cho, we did not find a meaningful trend besides that of NAA/Cr and NAA/Cho in the normal regions. Along with the small size of patient pool, this study harbors some limitations due to the retrospective nature of the analysis and the absence of age-matched controls. No data was obtained from healthy controls or asymptomatic-carrier relatives as ethical issues were hard to address, and there were realistic hurdles with cooperation and sedation in children, especially when studying an extremely rare disease. We would like to emphasize that the results should be interpreted with caution as this is a preliminary study with few comparable literature existing, and its statistical analysis being performed in an exploratory manner with no correction for further multiple comparisons. Another short coming of this study is the qualitative categorization of MRS results solely based on the neuroradiologist's interpretation, rather than quantitative methods such as computerized analysis. With a larger group of patients in the near future, hopefully this study may expand and validate its findings by applying conservative methods including correction for multiple comparisons and provide more insight on the use of MRS and serum biomarkers for monitoring symptomatic MELAS patients.

## Conclusion

We have shown that MRS is useful for monitoring changes in brain metabolites, particularly those associated with stroke-like episodes, which affect patient's functional ability and quality of life. Additionally, certain MRS parameter changes accompany changes in serum biomarkers, and their correlations change over the course of the disease. Our results suggest that, during the acute stage of stroke-like episodes, monitoring changes in both serum lactate and Lac/Pyr ratio is important, as these reflect changes in the MRS lactate peak. During the chronic stage, monitoring the serum Lac/Pyr ratio in relation to MRS lactate peak is important for monitoring the remaining respiratory chain enzyme defect. In regions without structural changes and during the clinically silent stage, monitoring serum lactate in accordance with MRS NAA-associated metabolites is important for observing the progress of the underlying neuronal disintegration. Thus, accurate interpretation of serum and MRS studies in relation to patient's clinical status can facilitate appropriate monitoring and care management of symptomatic MELAS patients, helping them to maintain a better quality of life and to prevent functional decline.

## Author contributions

HL, C-SY, and Y-ML designed the study protocol. HL collected data and wrote the first draft of the manuscript under the mentorship of C-SY and Y-ML. All coauthors critically reviewed the manuscript.

### Conflict of interest statement

The authors declare that the research was conducted in the absence of any commercial or financial relationships that could be construed as a potential conflict of interest. The reviewer LS and handling editor declared their shared affiliation at the time of the review.

## Abbreviations

Cho: choline
Cho/Cr: choline/creatine ratio
Cr: creatine
Lac/Pyr: lactate/pyruvate ratio
MELAS: mitochondrial encephalomyopathy, lactic acidosis and stroke-like episodes
MRI: magnetic resonance imaging
MRS: magnetic resonance spectroscopy
NAA: *N*-acetylaspartate
NAA/Cho: *N*-acetylaspartate/choline ratio
NAA/Cr: *N*-acetylaspartate/creatine ratio.
